# High Quality Graphene Thin Films Synthesized by Glow Discharge Method in A Chemical Vapor Deposition System Using Solid Carbon Source

**DOI:** 10.3390/ma13092026

**Published:** 2020-04-26

**Authors:** Le Wang, Jie Sun, Weiling Guo, Yibo Dong, Yiyang Xie, Fangzhu Xiong, Zaifa Du, Longfei Li, Jun Deng, Chen Xu

**Affiliations:** 1 Key Laboratory of Optoelectronics Technology, Beijing University of Technology, Beijing 100124, China; wangle316@emails.bjut.edu.cn (L.W.); guoweiling@bjut.edu.cn (W.G.); donyibo@emails.bjut.edu.cn (Y.D.); xieyiyang@bjut.edu.cn (Y.X.); fangzhuxiong@emails.bjut.edu.cn (F.X.); 17801011216@163.com (Z.D.); 18800153166@163.com (L.L.); dengsu@bjut.edu.cn (J.D.); xuchen58@bjut.edu.cn (C.X.); 2 National and Local United Engineering Laboratory of Flat Panel Display Technology, College of Physics and Information Engineering, Fuzhou University, Fuzhou 350100, China; 3 Fujian Science & Technology Innovation Laboratory for Optoelectronic Information of China, Fuzhou 350100, China

**Keywords:** graphene, glow discharge, graphite, chemical vapor deposition, metal catalyst, solid carbon source, plasma

## Abstract

Arc discharge is traditionally used to synthesize randomly arranged graphene flakes. In this paper, we substantially modify it into a glow discharge method so that the discharge current is much more reduced. The H_2_ and/or Ar plasma etching of the graphitic electrode (used to ignite the plasma) is hence much gentler, rendering it possible to grow graphene in thin film format. During the growth at a few mbar, there is no external carbon gas precursor introduced. The carbon atoms and/or carbon containing particles as a result of the plasma etching are emitted in the chamber, some of which undergo gas phase scattering and deposit onto the metallic catalyst substrates (Cu-Ni alloy thin films or Cu foils) as graphene sheets. It is found that high quality monolayer graphene can be synthesized on Cu foil at 900 °C. On Cu-Ni, under the same growth condition, somewhat more bilayer regions are observed. It is observed that the material quality is almost indifferent to the gas ratios, which makes the optimization of the deposition process relatively easy. Detailed study on the deposition procedure and the material characterization have been carried out. This work reveals the possibility of producing thin film graphene by a gas discharge based process, not only from fundamental point of view, but it also provides an alternative technique other than standard chemical vapor deposition to synthesize graphene that is compatible with the semiconductor planar process. As the process uses solid graphite as a source material that is rich in the crust, it is a facile and relatively cheap method to obtain high quality graphene thin films in this respect.

## 1. Introduction

Since its appearance, graphene has been applied to solar cells, sensors, composite materials, photocatalysis, and other fields due to its outstanding characteristics such as high carrier mobility, high mechanical strength, high specific surface area, high transmittance, and high thermal conductivity [1]. The graphene synthesis methods include mechanical exfoliation, chemical exfoliation, epitaxial graphene on SiC [2], molecular beam epitaxy (MBE) [3], chemical vapor deposition (CVD) [4], and arc discharge (AD) [5,6,7,8,9,10,11,12,13], etc. Among these technologies, mechanical and chemical exfoliation of graphite can only produce irregular shaped flakes or powders; epitaxial graphene on SiC is hardly transferable, very expensive, and the graphene film size is limited by the available SiC substrate; MBE graphene is not mature and the material quality is modest; only CVD technique can produce scalable graphene thin films that are compatible with standard semiconductor planar process. Regarding AD, because of the low controllability, it has not received widespread attention in graphene synthesis thus far. Traditionally, the AD method is used to synthesize fullerenes, single or multi-wall carbon nanotubes, randomly arranged graphene flakes, carbon nanoparticle-based light-emitting devices, etc. [5,6,7,8,9,10,11,12,13]. When making graphene by AD, the graphite electrode is usually used as the carbon source. Typically, the electrode can be completely consumed in just ten minutes or so due to the high electrical current and the intense etching reaction, and the graphene grown is multi-layered (about 2 to 10 layers) and very defective [5,6,7,8,9,10,11,12,13]. A variant version of the AD method is the synthesis of graphene by the so-called hydrogen AD exfoliation of graphite, which is often with the involvement of graphene oxides [13]. Nevertheless, it is also not very controllable and, most importantly, it is not compatible with today’s semiconductor processing and cannot produce graphene in the format of thin films. 

In this paper, we have substantially modified the traditional AD method, based on which we introduce a new graphene synthesis technique that is called the glow discharge (GD) deposition method. The equipment used in this work is actually a standard PECVD (plasma enhanced CVD) system that was not originally intended for the GD use. In the chamber, which is filled with H_2_ and/or Ar gas, we ignited the plasma, and it slightly etches the graphite electrode that is used to start the plasma. Because of this, we were able to grow graphene on the surface of metal catalysts even without using any carbon precursor gas. The possible deposition mechanism is explained by the plasma-based physical and/or chemical etching of the graphitic electrode, followed by gas phase scattering of the produced carbon atoms and carbon based particles, which are in part transported towards the catalytic surfaces of the metallic substrates situated on the heater. After the catalytic graphitization on the metal surface, the prepared graphene/metal is unloaded, and the graphene can be transferred to insulators using a wet etching-based technique [14,15]. The parameters of the GD method are systematically optimized, and the materials are characterized in detail. Compared with MBE, partly due to the existence of catalyst, the GD offers a better material quality, and the growth time is also much shorter. Even including the pumping and heating/cooling procedure, it only takes 20 min for one run. Compared to standard CVD, the GD offers an alternative method that negates the need for work on the precursor gas composition, the decomposition rate, and the large growth parameter space. Furthermore, the growth temperature of GD is somewhat lower. Compared to AD, the GD here is a much gentler process. Most importantly, the graphene prepared by GD is a continuous thin film with high quality, which can be transferred to insulators and used to make electronic devices. Our work explores the potential of the traditionally overlooked AD method in synthesizing graphene thin films that are compatible with semiconductor planar processing. Therefore, it is of value to scientists and engineers who work with the synthesis of graphene and its electronic device applications.

## 2. Experimental Procedures and Methods

In order to verify and benchmark the quality of the graphene obtained by the new GD graphene production mode proposed in this paper, we first grow standard graphene thin films by CVD using two kinds of conventional catalytic metals (Cu-Ni alloy film and copper foil) [16,17] as control samples. The CVD process is described in our earlier publication [14,15]. Those two types of metals are also the substrates that are used to grow graphene by our GD method, and their preparation methods are as follows. 

(1) Cu-Ni alloy thin films. After the Si wafer is cleaned by standard procedure, 300 nm SiO_2_ is grown by inductively coupled plasma chemical vapor deposition (ICP-CVD). This is because, later, the Cu-Ni will be removed and the grown graphene will “land” on the SiO_2_, and 300 nm silicon dioxide is known to facilitate the observation of graphene under an optical microscope after growth, due to the optical interference effect [18]. Magnetron sputtering is used to sputter Cu-Ni (2:1) alloys of different thicknesses on the SiO_2_/Si wafers to study the effect of catalyst thickness on the quality of graphene. 

(2) Copper foils. High purity polycrystalline copper foils are purchased commercially.

**Figure 1:**
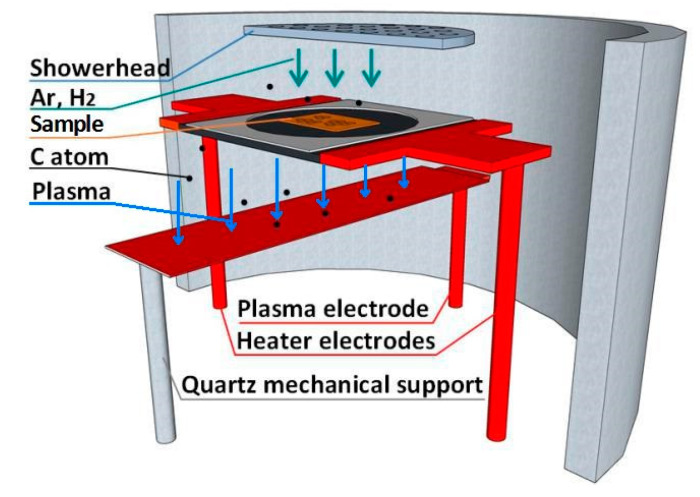
Schematic illustration of the growth chamber of the plasma enhanced chemical vapor deposition (PECVD) equipment which is used for the glow discharge (GD) synthesis of graphene in this work.

The growth system used in this work is a graphene PECVD of the model Black Magic, produced by Aixtron Nanoinstruments Ltd. The outwall of the growth chamber is made of stainless steel with a quartz shield as the inwall. As shown in Figure 1, gases come from the top via a quartz showerhead, which has small holes to distribute the gases uniformly to the samples below. The arrows indicate the direction of the gas flow. The samples are placed on a graphitic heater supported by two vertical metal rods which are also working as the heater electrodes. Alternating current (AC) current is sent through the two electrodes to heat the heater by Joule heating. On the heater, there is a ceramic clamp which can hold a maximum 2 inch sample. A third electrode, which we call the plasma electrode, is right below the heater (with ~4 cm distance). An AC or direct current (DC) voltage of several hundred volts can be applied to the plasma electrode, with respect to the potential of the heater in order to ignite the plasma (In this paper we have used the AC configuration).

We place the as-prepared Cu-Ni alloy thin film or copper foil samples on the graphite plate heater in the equipment (see Figure 1) for the GD growth of graphene. The copper foil sample, however, should be first put in a quartz bowl, because if the foil is in direct contact with the heater, the electrical current leaked into the copper from the graphite is hard to control, which will add extra heating and may melt the copper. After evacuating to a base pressure of 2 × 10^−3^ kPa, we introduce Ar and/or H_2_ gases (for example, with a ratio of 5:1) into the chamber, and the pressure reaches 5 × 10^−1^ kPa. The procedure is repeated three times and then the gas input is turned off. This is because we have found that without a flowing gas, the plasma power during the initiation is more stable than is the case with a gas flow. The heating rate is 5 °C/s, at which we increase the heater temperature to the desired growth temperature (typically 600–1000 °C). The samples are held at this temperature for ten minutes for annealing to increase the crystallinity of the metals [19], which is especially useful for the Cu-Ni alloy. Then, the plasma is ignited. The power intensity is 40 W, and the AC frequency is 20 kHz. After 5 min of growth, the plasma is turned off, and the temperature is cooled down to 500 °C at a rate of 5 °C/s. Afterwards, the Ar gas is introduced to the chamber to accelerate the temperature dropping until it is cooled to room temperature, and the samples are unloaded.

**Figure 2:**
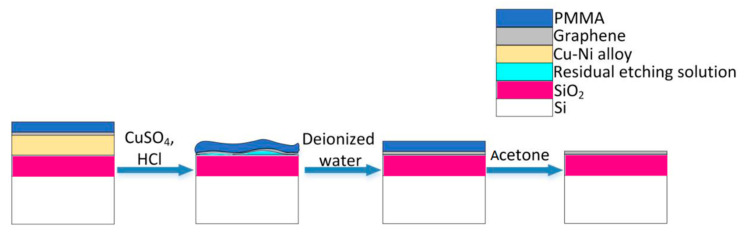
Process of “transferring” the graphene grown by Cu-Ni alloy onto its own SiO_2_/Si substrate.

After growth, the copper-nickel alloy and the copper foil samples are all spin-coated (4000 rpm for 30 s) with a layer of polymethyl methacrylate (PMMA) with about 70 nm thickness. Then, it is heated on a 150 °C hot plate for 10 min. A metal etching solution (CuSO_4_:HCl:H_2_O = 5 g:25 mL:50 mL) is prepared. The copper-nickel sample is placed at the bottom of the beaker, whereas the copper foil sample is floating on the surface of the solution. The copper foil grown graphene is transferred to another 300 nm SiO_2_/Si substrate by the standard wet transfer process [14,15]. The graphene grown on the Cu-Ni alloys, however, is transferred to its own SiO_2_/Si substrate through the etching mechanism that is shown in Figure 2. The chemical solution penetrates the PMMA and etches the metal beneath. Because of the buffering of the PMMA layer, the reaction will become very gentle, which reduces the possibility of graphene being damaged by the etching process [17]. After etching, we put the PMMA/graphene/SiO_2_/Si complex in deionized water for 30 min to clean up the residual chemicals. In order to improve the adhesion between the graphene and the substrate, it is baked at 150 °C for 15 min on a hot plate. Then, it is placed in acetone for 1 h to remove the PMMA from graphene. Finally, the sample is placed in a ventilated place for 10 min to dry the acetone. During the experiment, nevertheless, after the metal etching the van der Waals force between the SiO_2_/Si substrate and the PMMA/graphene is relatively weak, and the rinsing process in deionized water might cause the PMMA/graphene to float on the surface of solution. Applying a little PMMA to the edges of the sample helps hold the film, and can increase the success rate of the graphene transfer.

After the sample preparation, we use Raman spectroscopy with the excitation wavelength at 532 nm to characterize the quality of the graphene thin films, as well as to determine the number of graphene layers grown with different metal catalysts. Raman mapping is also performed. A scanning electron microscope (SEM, MERLIN Compact, Zeiss, Oberkochen, Germany) is used to examine the morphology of the metals after the annealing and after the graphene deposition.

## 3. Results and Discussion

**Figure 3:**
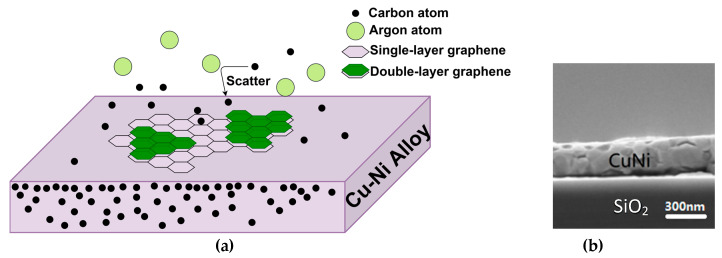
(**a**) Schematic diagram of the deposition of graphene by GD method on the Cu-Ni alloy. The carbon atoms resulted from the plasma etching of the graphite electrode are transported to the metal catalytic surface via scattering. (**b**) Scanning electron microscope (SEM) image (cross-sectional view) of the Cu-Ni alloy after annealing at 900 °C.

The schematic illustration of the graphene deposition procedure is shown in Figure 3a. The production mechanism of graphene involves two steps: (1) the creation of carbon atoms or carbon containing particles; (2) the graphitization of these particles. Regarding the graphitization on the substrate, it is the same for both the GD growth method and the normal graphene CVD. However, in a regular CVD, the formation of carbon atoms or carbon containing particles is via the decomposition of hydrocarbons, whereas in GD it is through the gentle plasma etching of the electrode. The possible carbon particle formation mechanism of our GD method is explained as follows. GD and AD are two typical discharge procedures of gaseous species. Compared to the traditional AD method, the process in our GD is much gentler. Usually, GD occurs at a higher and more stable voltage, but the current is smaller (in mA). AD happens at lower voltage (typically 1~10 V), but the current is in ampere range (typically 10~100 A). It's often much brighter and hotter compared to GD. In our machine, the applied plasma voltage is in the order of several 100 volts, the current is 0.1–0.5 A, and the pressure is a few mbar. These are typical conditions for GD but not for AD [20]. Under our condition, therefore, the phenomenon should be defined as GD instead of AD, which is a lot gentler. The plasma etches the graphite, but does not exfoliate it like in the AD. The emitted carbon atoms and carbon containing particles are graphitized into textured thin films on the metal surfaces. That eliminates the possibility to produce random graphene flakes as the AD method does. We have not found any reports about graphene thin films produced by AD. Therefore, the graphene produced by AD cannot be applied in electronic chips. It is mainly for other applications such as composite materials. In our GD, on the other hand, as shown later, no matter what Ar to H_2_ gas ratios are used, we can always obtain thin film graphene conformally coating on the metal catalysts, provided that other conditions are optimized (e.g., temperature, pressure, plasma power). Since there is no carbon containing gas introduced in the machine, the only explanation is that the graphene grows from the etched graphite. Note that we have found the graphene thin films do not form when no plasma is present. Thus, we can conclusively exclude the possibility that the graphene’s carbon source comes from unintentionally introduced carbon species in the chamber such as carbon contamination, oil vapor from the pump, etc. In our experiment, the etching mechanisms of H_2_ and Ar plasmas are different. The former (H_2_ plasma) is mainly a chemical process, where the etching of graphite is achieved through hydrogenation (forming C-H bonds) and the subsequent releasing of hydrocarbons such as CH_4_ [21]. Some authors, however, suggest a slightly different mechanism, where the hydrocarbon formation is a result of first ionic bombardment and subsequent chemical reaction [22]. The latter (Ar plasma) is basically a physical process, where the etching is simply an ionic bombardment and creates carbon atoms [23,24]. So, how can those carbon atoms or carbon-based particles that resulted from the etched graphite turn into graphene thin films? When the plasma electrode is etched, the produced particles are transported onto the catalytic metal substrates via gas phase scattering. The mean free path *λ* of the particles can be approximately estimated by the ideal gas formula $\lambda =\frac{1}{\sqrt{2}\pi d^{2}n}$, where *d* is the effective diameter of the particle, and *n* is the number of particles per unit volume. Using that equation, the mean free path of the carbon-based particles is in the order of a few millimeters, which is much shorter than the distance between the plasma electrode and the samples on the heater (~4 cm). Therefore, the particles undergo many scatterings in the chamber and some particles will have chances to be directed toward the samples, as depicted in Figure 3a. In fact, since the graphite heater is also immersed in the plasma, it can be slightly etched, and supply some of the carbon particles as well. Because of the existence of the catalysts, graphitization happens on their flat surfaces and graphene thin films grow therein.

The introduction of the GD production mode in the PECVD chamber makes it feasible to grow thin film graphene under our conditions, which is not possible in the traditional AD method, which can only produce chaotic flakes. That is a new feature in graphene synthesis by gas phase discharge, both from fundamental and application points of view. Furthermore, in the GD method, the graphene is grown from cheap graphite and not an expensive carbon precursor gas, reducing the cost in this regard. Also, because there is no carbon precursor gas, it makes the process optimization much easier, without the need to monitor the precursor gas ratios. Only H_2_ and/or Ar gases are required in the chamber. As can be seen later, the quality of graphene is insensitive to the H_2_ to Ar ratio. According to our experiment, the material quality can be optimized by adjusting parameters such as the type of catalytic metals and the deposition temperature. Finally, after the GD growth, we have measured the 300 μm thick plasma electrode and could not detect any weight loss. Therefore, the process is indeed very gentle, and the consumption of the graphite material is tiny. In our practice, the same graphite electrode can be used for hundreds of runs.

**Figure 4:**
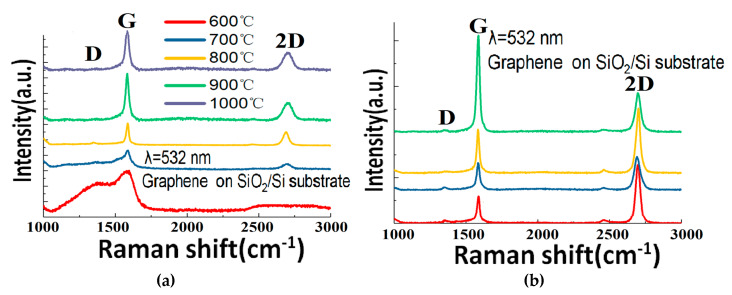
(**a**) Raman spectra of the graphene grown at different temperatures on Cu-Ni alloy and transferred to SiO_2_/Si substrates. (**b**) Raman spectra captured at different positions in the same graphene sheet grown on a Cu-Ni alloy at 900 °C and transferred to its SiO_2_/Si substrate.

In Figure 3b, the cross-sectional morphology of the Cu-Ni alloy after annealing can be seen. The Cu-Ni thin film thickness is 300 nm. It can be seen that the film is rather flat, and the copper and nickel have been uniformly alloyed after the annealing at 900 °C. The Raman results of the Cu-Ni alloy grown graphene samples can be seen in Figure 4a. The G band appears in each curve as a typical signature of a sp^2^ hybridized graphitic carbon structure. As the temperature rises from 600 °C to 1000 °C, the characteristic 2D peaks of the graphene Raman spectroscopy gradually appear. Meanwhile, the D peak eventually decreases. These features indicate that the number of defects or disorders in the graphene decreases. The Raman characterization results of the samples at 800 °C–1000 °C are more or less similar to each other. The Raman results are also comparable with standard CVD graphene grown in the same type of machine (see our previous publications [14,15]), which confirms the crystalline quality of the as-grown graphene. The feasibility of using the GD method to grow reasonably high-quality graphene with catalyst at around 900 °C is thus experimentally proved.

In order to examine the structural uniformity of the graphene along the sample, more detailed characterizations are carried out. Figure 4b shows the Raman spectra measured at different positions of the same graphene sheet grown at 900 °C on a Cu-Ni alloy and transferred to its SiO_2_/Si substrates. It is well established that the number of layers in graphene with good crystallinity can be estimated through the 2D/G ratio, and the shape and width of the peaks [25]. It can be seen that the number of graphene layers grown by the Cu-Ni alloy varies between monolayer, bilayer, and multilayers at different locations across the surface of the substrate. This is explained by the carbon segregation mechanism during the graphene growth on the metal [26], because the nickel content in the alloy has a high carbon solubility of 1.26 at.% [27] (see Figure 3a). Apart from the surface catalysis process, the dissolved carbon species can emit to the surface of the alloy upon cooling down, as a result of the reduced carbon solubility at lower temperature. Subsequently, at some areas the graphene layers are thicker due to the carbon segregation. If Cu is used as the catalyst, then the dominant growth mechanism is a surface catalytic graphitization procedure, because the carbon solubility is very low (only 0.0027 at.% [27]). Therefore, we have also grown graphene on copper foils without any Ni content, in order to obtain a large monolayer ratio, as will be shown thereinafter. 

**Figure 5:**
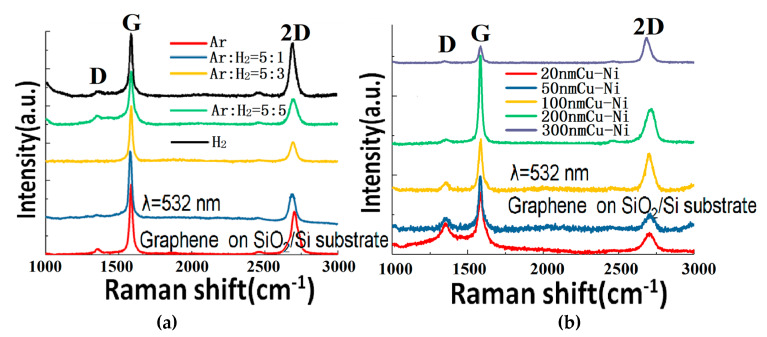
(**a**) Raman spectra of the graphene thin films grown with different gas ratios at 900 °C on 300 nm Cu-Ni and transferred to SiO_2_/Si substrates. (**b**) Raman spectra of the graphene grown at 900 °C (transferred to SiO_2_/Si) and Ar:H_2_ (5:1) gas ratio with different thicknesses (from 20 to 300 nm) of Cu-Ni alloy

Figure 5a shows the Raman spectra of graphene samples (transferred to SiO_2_/Si) grown on a 300 nm Cu-Ni alloy with different gas ratios at 900 °C. The gas mixture is varied from pure Ar, Ar:H_2_ = 5:1, Ar:H_2_ = 5:3, Ar:H_2_ = 5:5, to pure H_2_. It can be seen that the graphene quality almost does not change, which proves that the GD mechanism does not depend much on the Ar-H_2_ composition of the gas. No matter whether it is a large atom Ar gas or a small molecule H_2_ gas, the GD can etch the graphite electrode and supply carbon source anyway (although the etching mechanisms are different). Therefore, in future experiments, cheaper gas can be selected to prepare the graphene by this method. However, because the reaction temperature is typically above 800 °C, oxygen or air cannot be selected as the plasma gas so as to avoid the burning of the graphite.

Figure 5b shows the Raman spectra of the graphene (transferred to SiO_2_/Si) grown at 900 °C with a different thicknesses (from 20 to 300 nm) of Cu-Ni alloy and an Ar:H_2_ = 5:1 gas ratio. The original idea of this experiment was to reduce the amount of absorbed carbon (hence reducing the number of produced graphene layers) through reducing the thickness of the Cu-Ni alloy. However, the experimental results show that even if the 20 nm Cu-Ni alloy is used, it still can produce bilayer and multilayer graphene. On the other hand, as the thickness decreases, the D peak rises and more defects appear. This article considers that the graphene quality gets worse at reducing thickness of the alloy because during the growth, the metals of the 20 nm and 50 nm samples sublimate from the surface under high temperature and low pressure conditions, which gradually leads to the loss of the graphene catalysis effect. If we continue to test the growth on even thinner metals, the graphene quality will be even worse, and the alloy will melt due the lowering of the melting point with the reduction of the thickness. Towards the other end, when the thickness of the Cu-Ni alloy reaches 100 nm and above, the quality of graphene has gradually stabilized.

**Figure 6:**
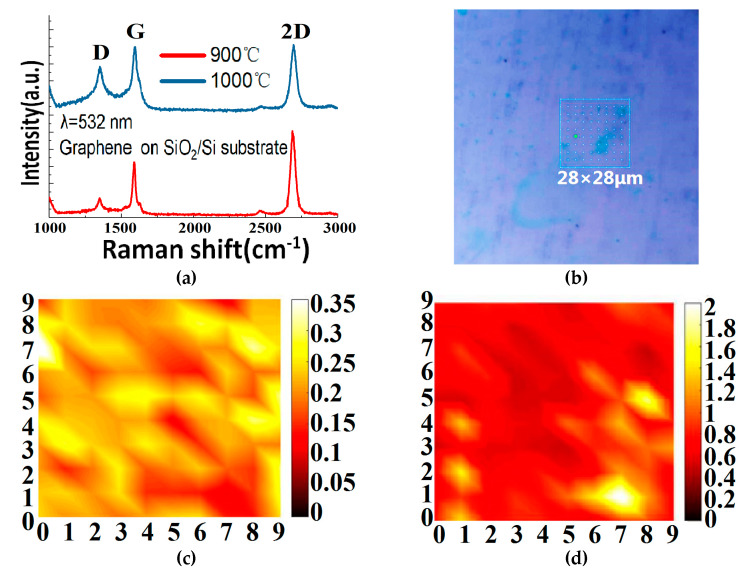
(**a**) Raman spectra of the graphene prepared by the GD method at 900 °C and 1000 °C by using copper foil as a catalyst. (**b**) Optical image (taken by Raman microscope) of a graphene thin film grown on copper foil at 900 °C and transferred to its SiO_2_/Si substrate. The 28 μm × 28 μm part indicated by the square is the area for Raman mapping. (**c**,**d**) Raman mapping (28 μm × 28 μm) of the D/G and G/2D ratios of the graphene grown on copper foil at 900 °C and transferred to its SiO_2_/Si substrate. In each image, there are 10 × 10 measured points and the color bar indicates the ratio.

**Figure 7:**
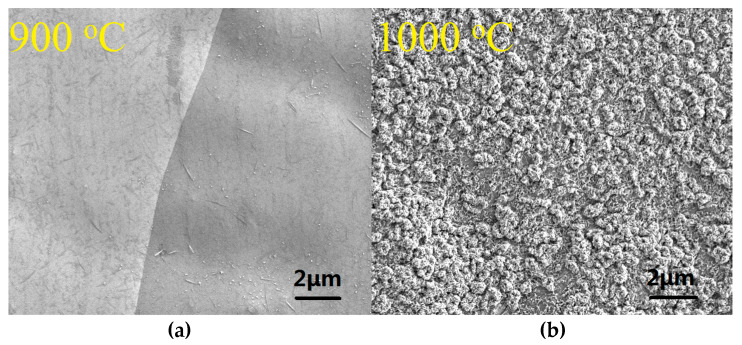
SEM images of the copper foils after coating with graphene at (**a**) 900 °C and (**b**) 1000 °C.

**Figure 8:**
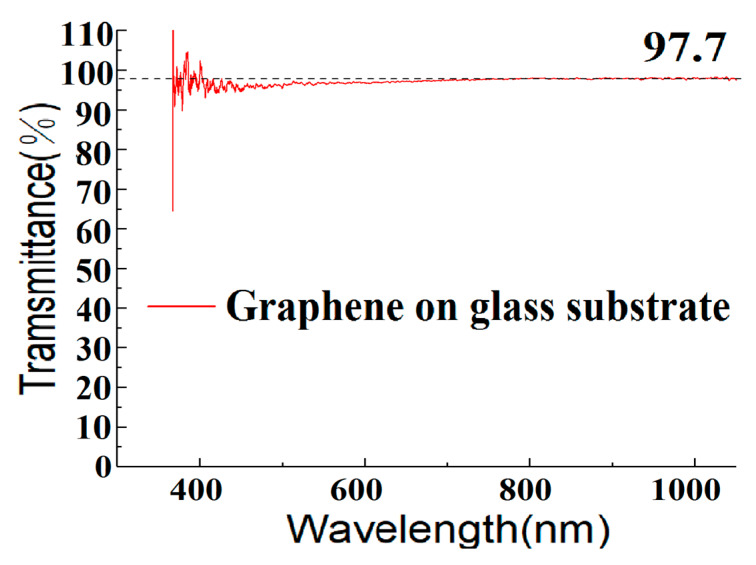
Optical transmittance of the as-grown graphene grown at 900 °C on Cu foil and transferred onto a glass substrate.

As indicated earlier, we have also grown graphene on copper foils by the GD method in order to boost the monolayer ratio. The corresponding Raman measurement results are demonstrated in Figure 6. From Figure 6a, we can see that, by using the GD method, we can grow high-quality single-layer graphene on copper foil at 900 °C. When the growth temperature is increased to 1000 °C, however, some low quality and—most likely—bilayer graphene is seen to grow, and where the 2D/G ratio decreases, the D peak rises and the number of defects increases. The reason is not yet clear, but this paper believes it could be explained as follows. Compared with the copper foil at 900 °C, the surface of the copper foil at 1000 °C becomes rougher (see Figure 7) because it is closer to Cu’s melting point 1083 °C. Eventually, the graphene quality deteriorates and some bilayer graphene starts to show up. The graphene quality is also worse compared with the graphene grown by the GD method on Cu-Ni at the same temperature (see Figure 5a). The effect is attributed to the fact that in graphene growth Ni has a much higher catalytic ability than Cu [28]. Figure 6b–d shows the Raman mapping data measured in a 28 µm × 28 µm (10 × 10 points) graphene area grown on copper foil at 900 °C and transferred to its SiO_2_/Si substrate. The ratio of I_D_ to I_G_ is less than 0.35, and the ratio of I_G_/I_2D_ is mostly around 0.5. This proves that the uniformity of the number of graphene layers is very good across the sample and the graphene is mainly single layer. Figure 8 shows the optical transmittance of the graphene grown at 900 °C and then transferred to the glass substrate. It has a transmission rate of about 97.7% in almost the full wavelength band, in agreement with the expected value for standard monolayer graphene. Those results confirm that the GD technique proposed in this paper performs excellently during the graphene growth catalysis on copper foils at 900 °C.

## 4. Conclusions

In this paper, we report a new type of graphene synthesis technology called GD deposition, using a standard PECVD machine. Compared to the traditional AD method, the GD technique uses a much smaller current, and the etching process of the graphite electrode that is used to ignite plasma is very gentle. The graphite electrode can be directly used as the carbon source for the graphene growth in a H_2_ and/or Ar plasma environment, and high-quality graphene thin films can be synthesized at about 900 °C on catalytic metals e.g., Cu-Ni alloy and pure Cu. The graphene production mechanism is a plasma etching of the graphite, followed by a carbon containing particle emission, where the particles are scattered towards the catalytic metal surfaces for graphene thin film formation. To our knowledge, this graphene production mode has never been reported before. It reveals that graphene sheets (not irregular flakes) can be obtained by gaseous discharge-based technology. The as-grown graphene is transferrable, and is characterized by Raman spectroscopy, SEM, and other means to prove the feasibility of the GD. It is found that the major factors during the graphene synthesis that can determine the material quality are the growth temperature and the type of catalytic metals, but not the gas ratios. This work shows that the GD graphene production model can be used to prepare graphene thin films without using carbon-containing gases such as methane and ethylene, which provides a new technology and a new insight for graphene synthesis. In future work, we will continue to improve the quality of graphene and increase the sample size. We will apply the graphene in semiconductor devices, such as current spreading layers of light-emitting diodes [29]. We will also adjust the experimental conditions and explore the interesting topic, whether the GD method can produce vertical graphene [30] or carbon nanotubes.
